# Supplementary material for: Towards the Development of an Insulin Degradation Test
Source: J Diabetes Res. 2026 Apr 29;2026:8533644. doi: 10.1155/jdr/8533644 (PMC13129213; doi:10.1155/jdr/8533644)
Supplement: Supplementary file 1 — Supporting Information 1 "Towards the development of an insulin degradation test" Supporting Figures.docx provides supplemental Figures S1–S9 that support the main text. [file JDR-2026-8533644-s002.docx]

**Supplemental Figures**

**Towards the development of an insulin degradation test**

**David Ritz^1^, Elizabeth-Lauren Stevenson^2^, Daniel Schultz^1^**

^1^ Department of Microbiology & Immunology – Geisel School of Medicine at Dartmouth.

^2^ Department of Molecular and Systems Biology – Dartmouth College. Hanover, NH 03755, USA.

**
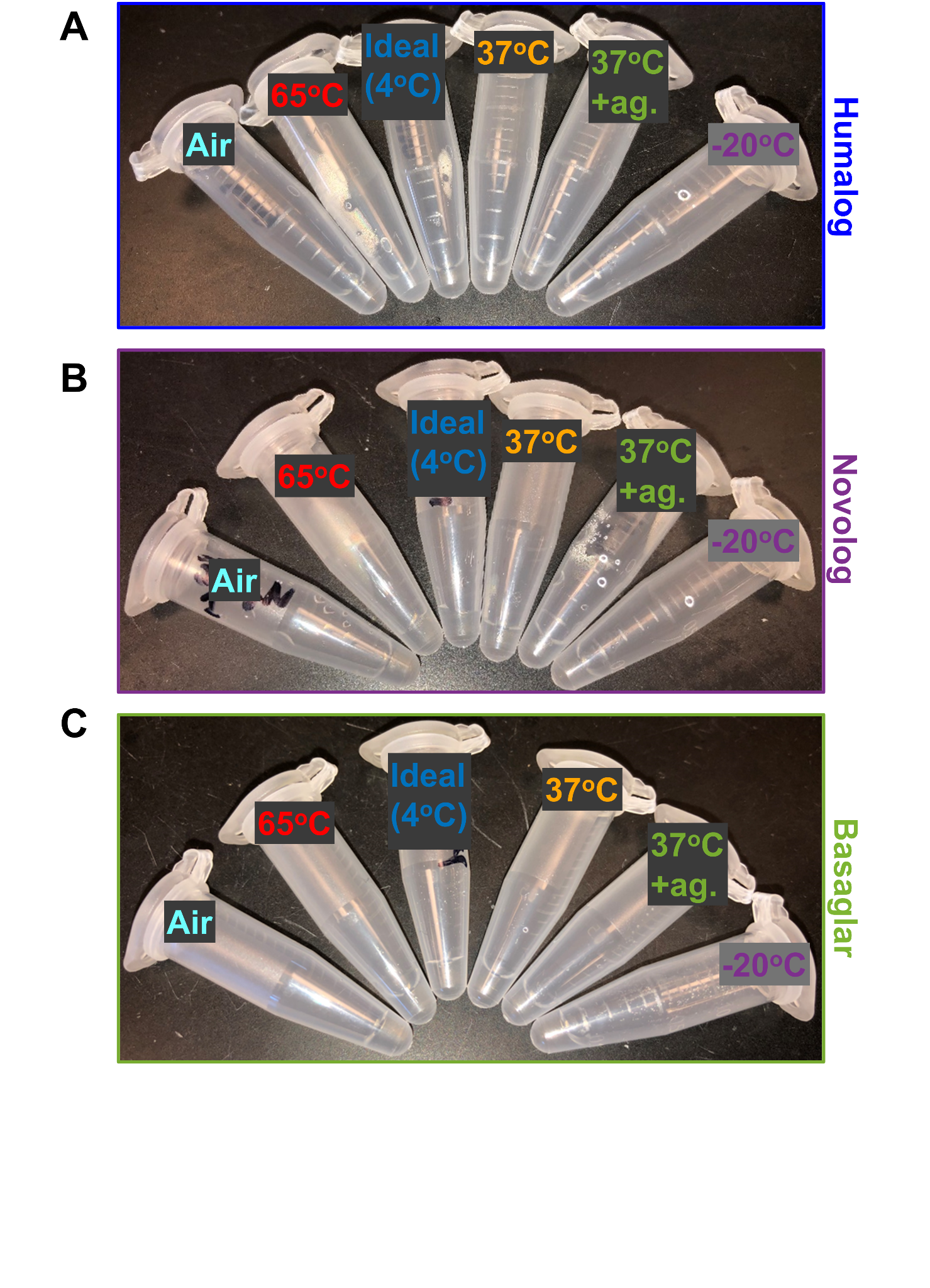
**

**Figure S1: Insulin rarely turns cloudy from acute degradations.** Representative images of **(A)** Humalog, **(B)** Novolog, and **(C)** Basaglar insulin samples in microcentrifuge tubes after 96 hours of degradation in each of the labeled stress conditions. UV and agitation samples are not shown.


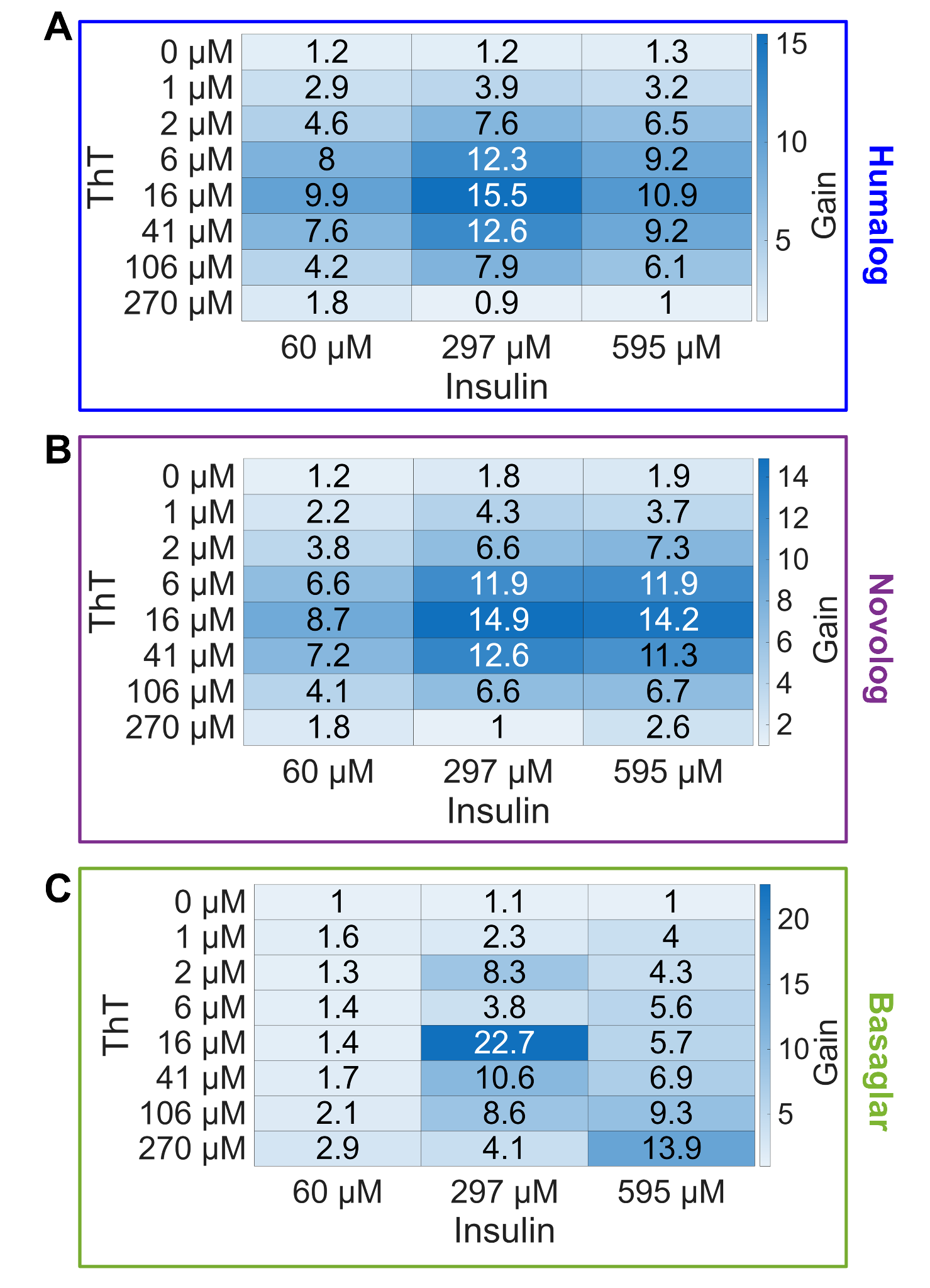


**Figure S2: Optimal ThT and insulin concentrations used during ThT assay.** Heat maps of ThT fluorescence gain for **(A)** Humalog, **(B)** Novolog, and **(C)** Basaglar fibrillated insulin samples relative to fresh insulin samples. To generate the fibrillated samples, Humalog and Novolog were exposed to 65°C for 24 hours and Basaglar was exposed to 37°C with agitation for 4 days. Because of these results, all ThT assays used an insulin concentration of 297 µM and a ThT concentration of 16 µM.


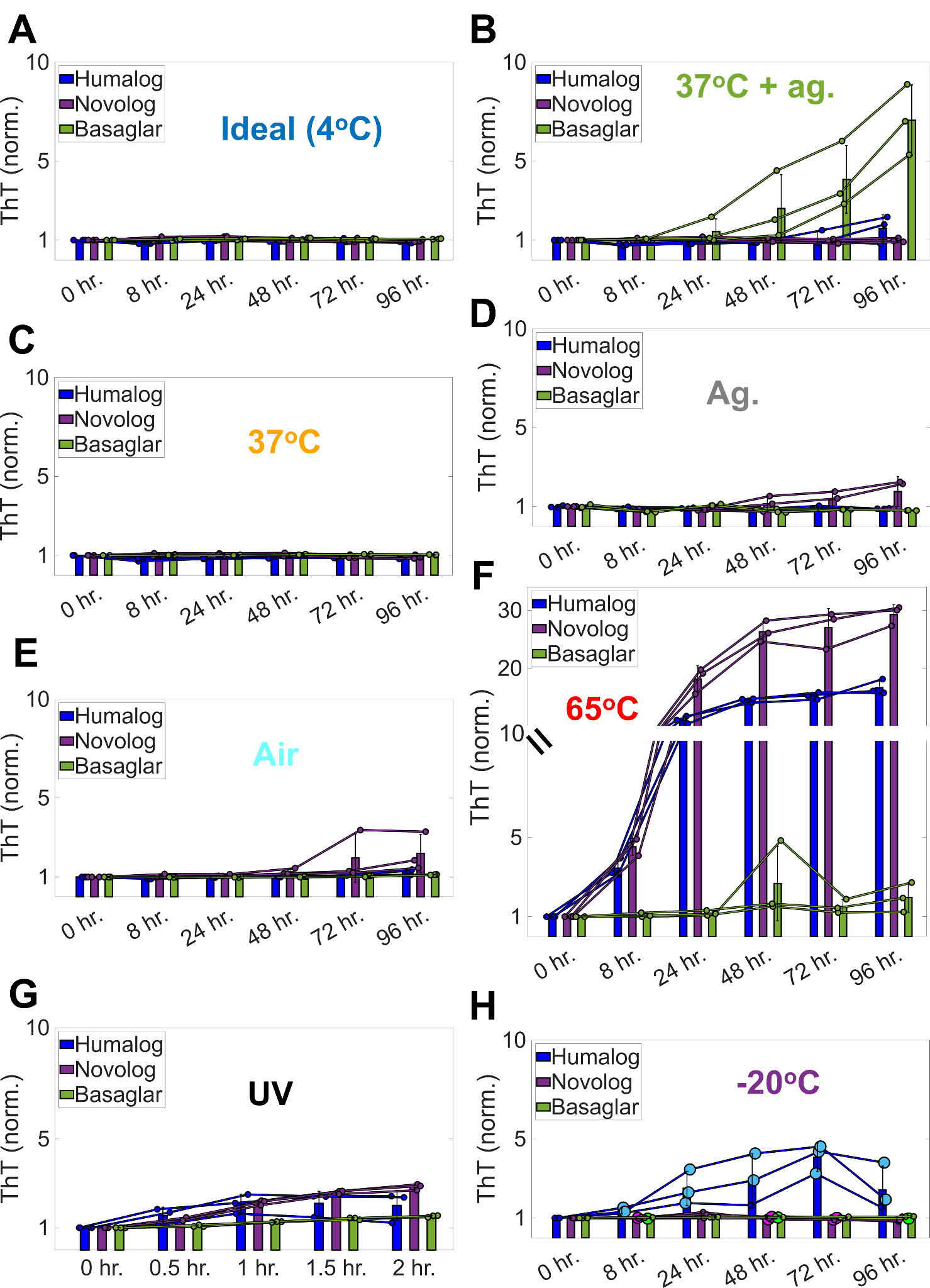


**Figure S3: Kinetics of fibril protein formation differ by stressor type and insulin type.** The kinetics of fibril protein formation was measured by ThT assay and tested for three aliquots of each insulin type when continuously exposed to: **(A)** ideal conditions at 4°C, **(B)** 37°C with agitation, **(C)** 37°C, **(D)** agitation, **(E)** air, **(F)** 65°C, **(G)** 302 nm UV light, and **(H)** -20°C. The large dots in panel H correspond to when the insulin went through a freeze-thaw cycle before sampling for the ThT assay. This was denoted because the insulin did not always freeze when in the -20°C freezer. The ThT fluorescence at the end of each experiment is displayed in **Fig. 1B**.


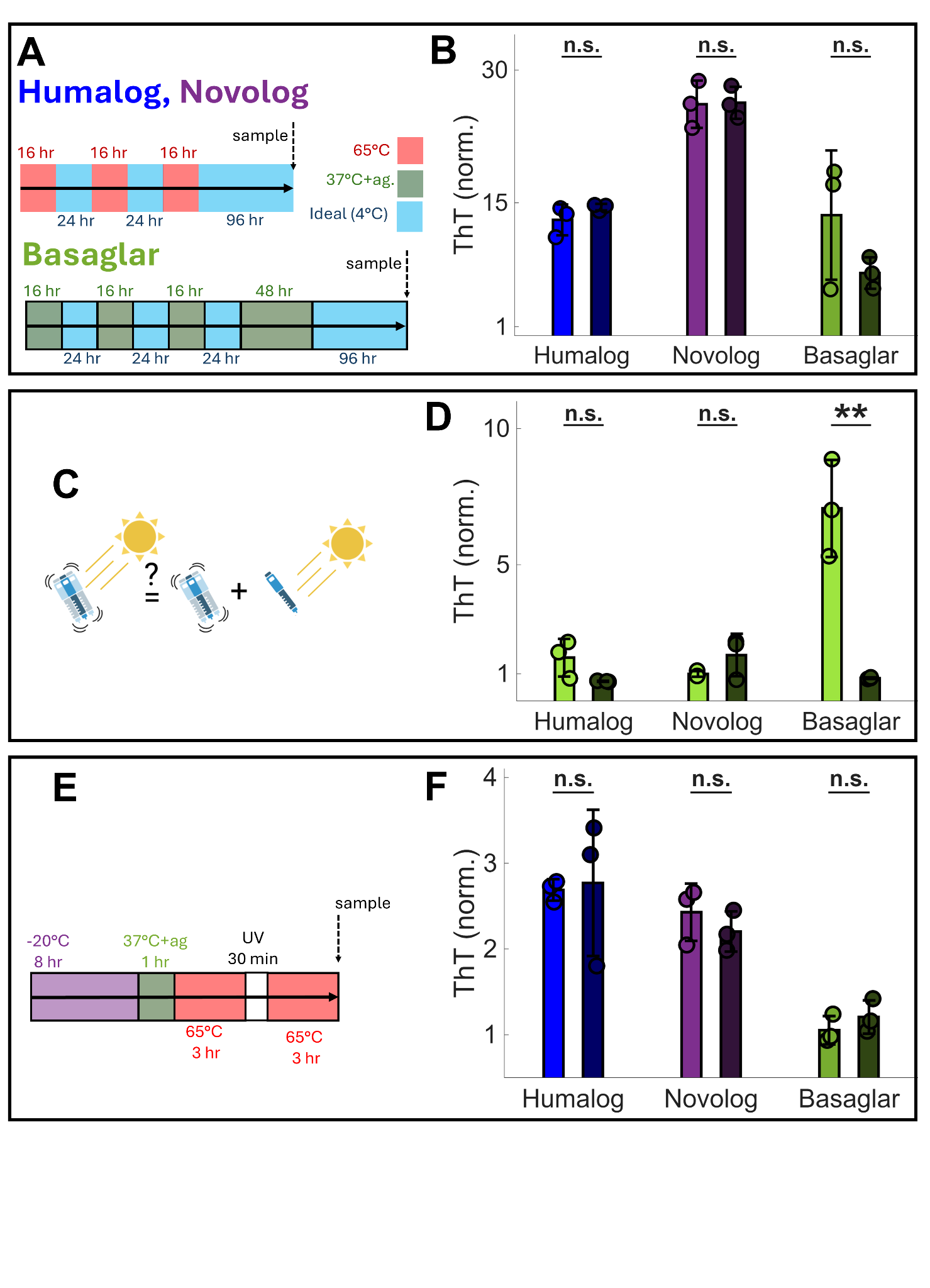


**Figure S4: Insulin fibrillation is generally additive and irreversible, but Basaglar 37°C with agitation is not additive.** **(A-B)** To assess the reversibility of fibrillation, each insulin type was exposed to rounds of a fibrillating stressor and was placed in ideal conditions at the end of each round. The lighter bar represents the observed ThT-reactive fibrils at the end of experimentation, while the dark bar represents the expected fibrillation from an additive model, calculated by adding the fibrillation of singular stressors by ThT assay. A sign of fibrillation reversibility would be a decrease in ThT-reactive fibril protein formation relative to the additive model. **(C-D)** Insulin was agitated while at 37°C, and its ThT-reactive fibrillation was compared to the same insulin separately agitated and then exposed to 37°C. The lighter bar represents the observed fibrillation when insulin is exposed to both stressors simultaneously, while the darker bar represents the expected fibrillation from an additive model, calculated by adding the fibrillation of singular stressors by ThT assay **(E-F)**. Insulin was exposed to rounds of freezing temperatures, hot temperatures, UV light, and agitation, denoted as the “Bad Beach Day” trial (BBD) in **Supplemental Data 1**. The lighter bar represents the observed fibrillation by ThT assay at the end of experimentation, while the darker bar represents the expected fibrillation from an additive model, calculated by adding the fibrillation of singular stressors by ThT assay. For each experiment, three replicates were used for each trial. In each panel, the observed degradation was compared to the expected fibrillation by a two-way t-test. The statistics notation denote ***** p < 10^-5^, **** p < 10^-4^, *** p < 10^-3^, ** p < 10^-2^, and * p < 5*10^-2^, and n.s. > 5*10^-2^.


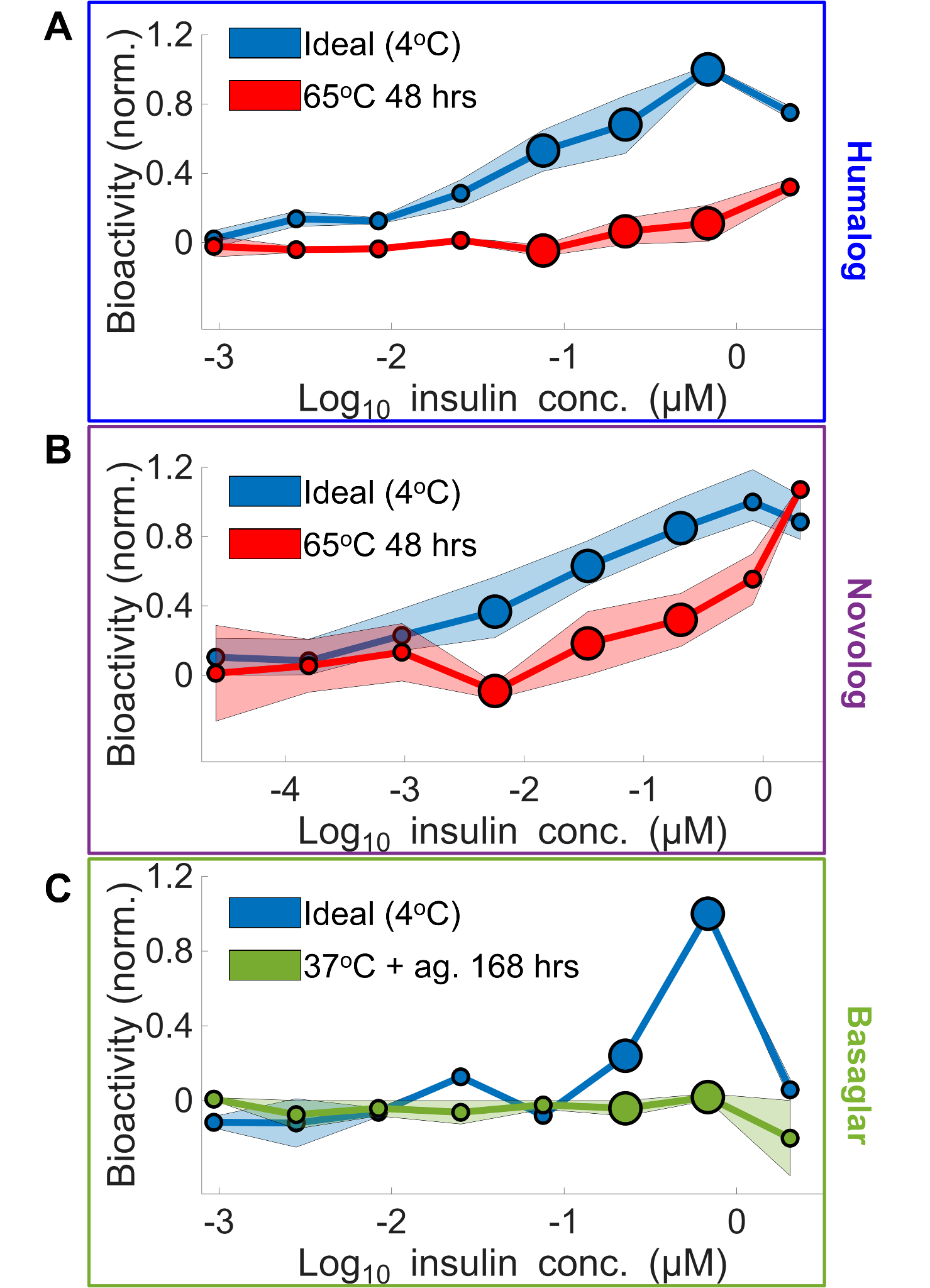


**Figure S5: CHO assay reveals differences in bioactivity between fresh and fibrillated insulin at certain insulin dilutions.** Fresh insulin and fibrillated insulin’s CHO bioactivity were compared for **(A)** Humalog, **(B)** Novolog, and **(C)** Basaglar. Large dots represent insulin concentrations that show large separations in bioactivity between fresh insulin and fibril-rich insulin by ThT assay and are near the EC_50_ value of fresh insulin, which is the insulin concentration causing a 50% bioactivity response. These concentrations were used for further analysis in the CHO bioactivity assay shown in **Fig. 2**. The shading around each line graph represents the maximum and minimum of two to three replicates.

**
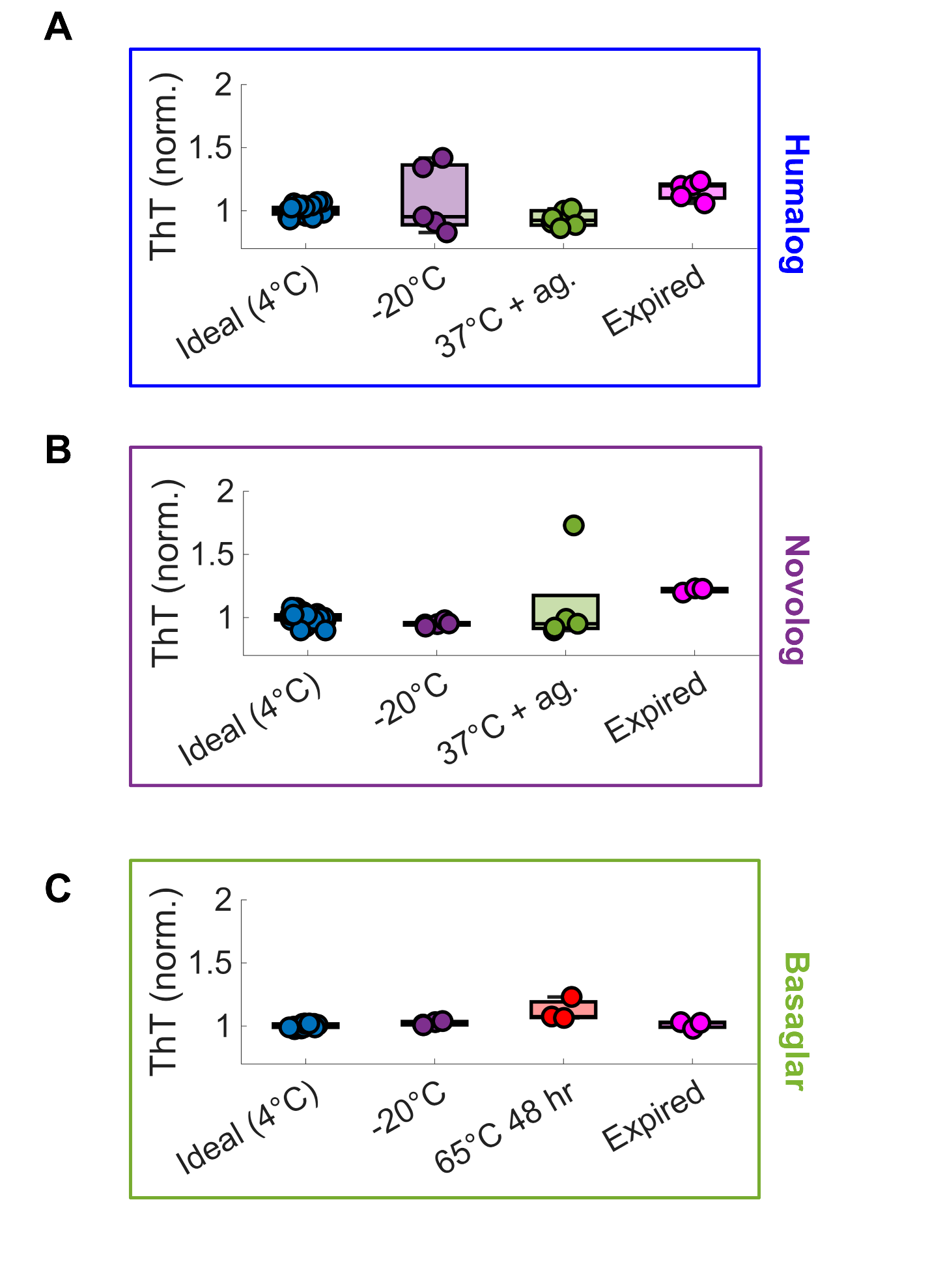
**

**Figure S6: Stressors can produce small changes to insulin fibril protein concentration.** During the CHO experiment, stressors for **(A)** Humalog, **(B)** Novolog, and **(C)** Basaglar were chosen that produce small or no changes to ThT fluorescence (**Fig. S3**). All trials were compared to their respective insulin analog at 4°C using ANOVA with Dunnett correction. This Figure was created by rescaling the axes of **Fig. 2C**, **Fig. 2F**, and **Fig. 2I**.


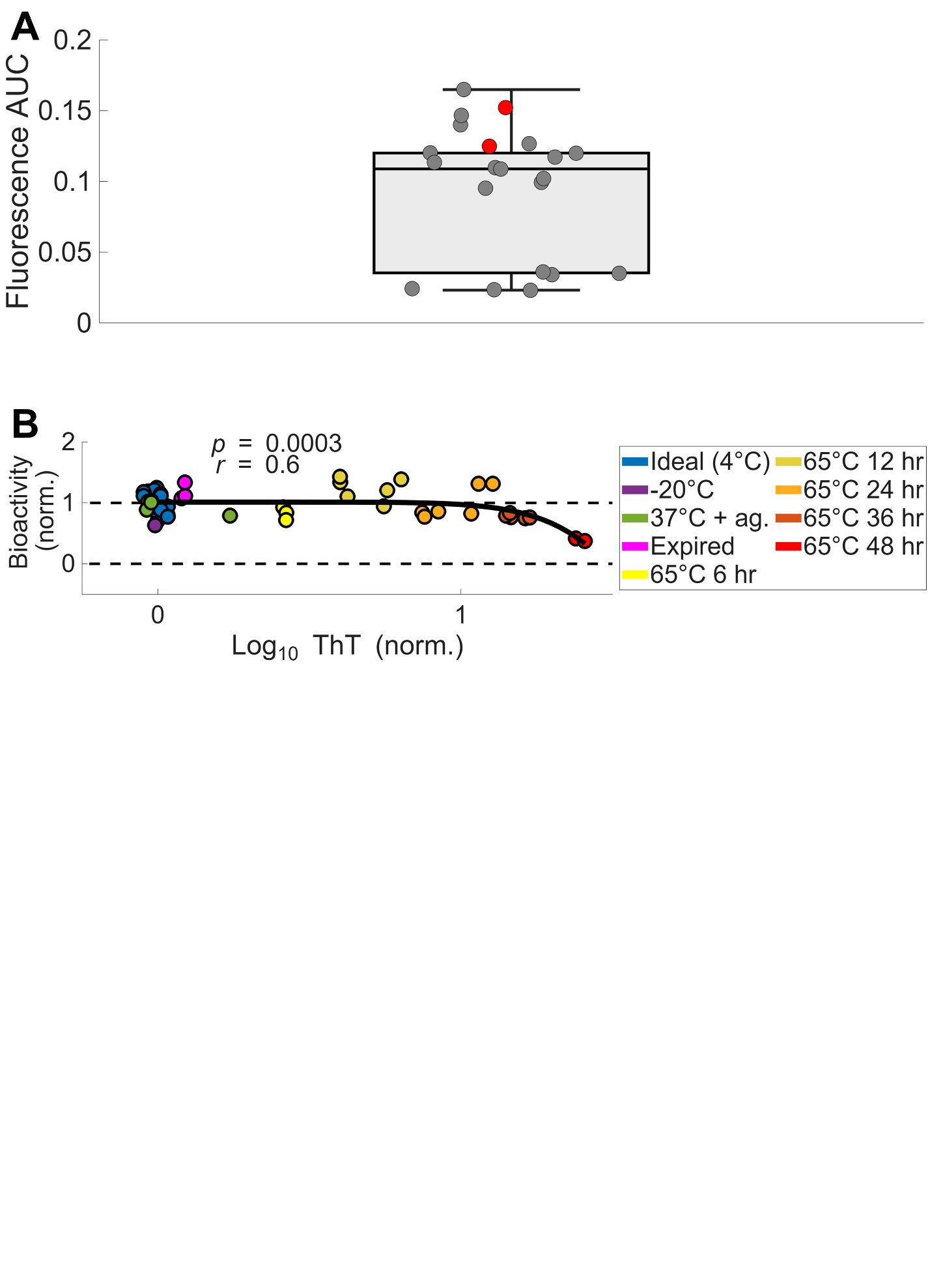


**Figure S7: Highly fibrillated and diluted Novolog insulin is an appropriate background measurement.** **(A)** Boxplot of AUC values across all plates using PBS-treated wells as background (gray), with individual PBS measurements shown as gray points. Overlaid are the AUC values using the lowest dilutions of Novolog incubated at 65°C for 48 hours (red, **Fig. S5B**), exhibiting similarly low signals. Therefore, these samples serve as surrogate background measurements for the Novolog CHO plate lacking PBS-treated CHO cells. **(B)** CHO bioactivity versus ThT fluorescence for Novolog with the surrogate-background plate removed. All CHO plates are included in **Fig. 2D**, showing only minor differences with **Fig. S7B**. This confirms that use of the surrogate background minimally affects the results.


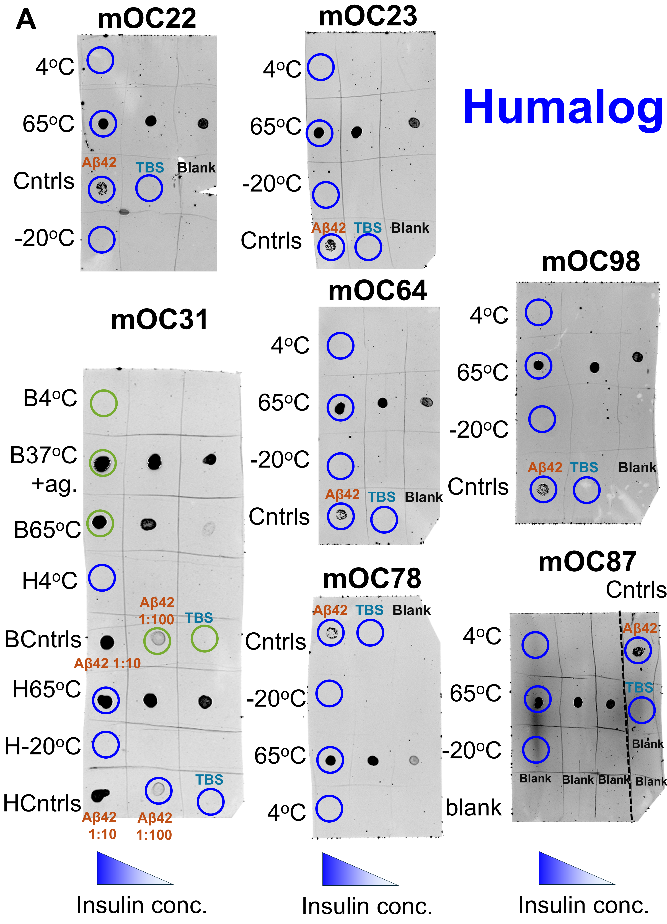


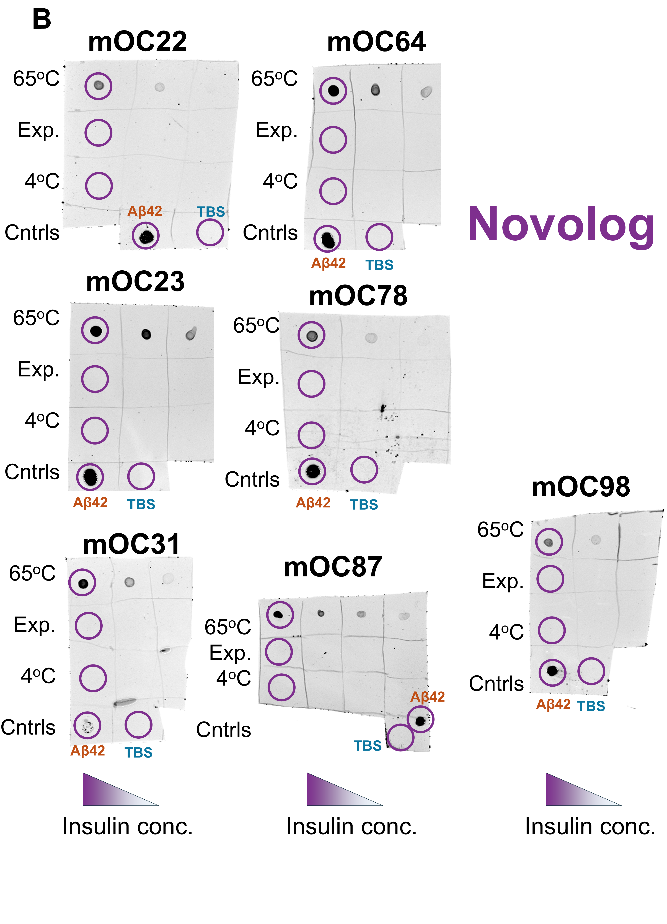


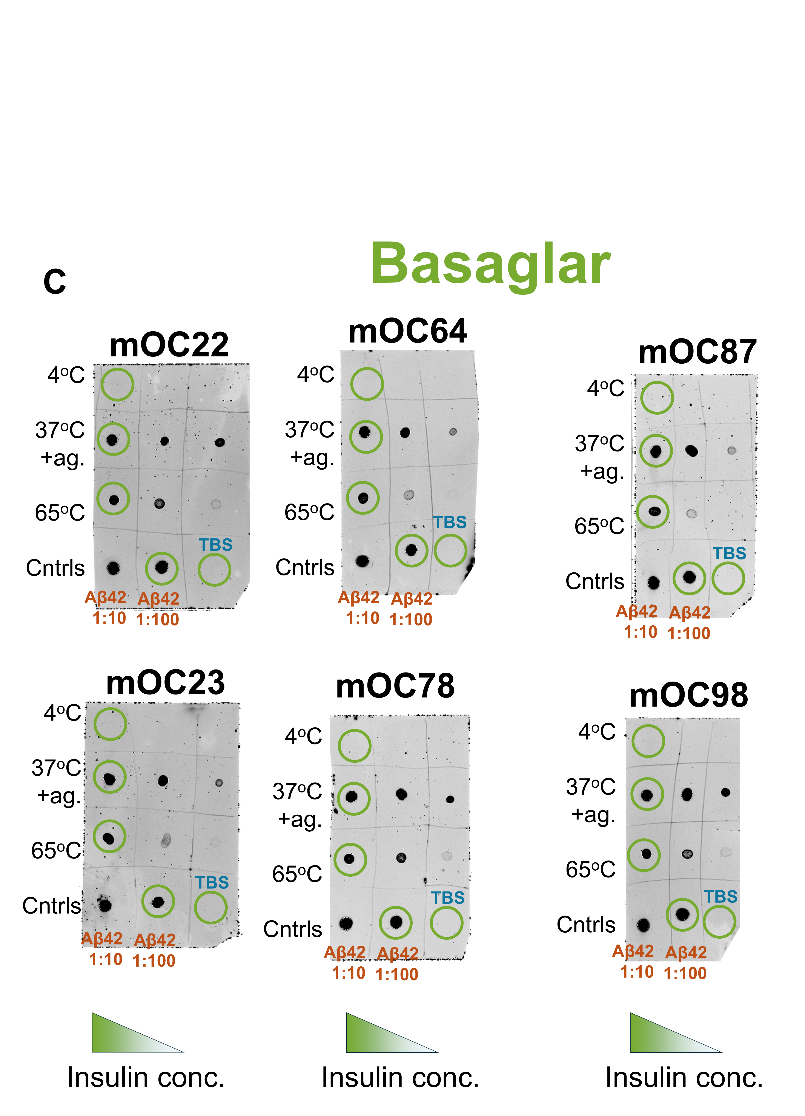


**Figure S8: Antibodies differ in their affinity to fibril proteins by stressor type. (A)** Humalog, **(B)** Novolog, and **(C)** Basaglar dot blots were conducted with antibodies that recognize general epitopes of fibril’s secondary structure and with insulin that were separately exposed to three different stressors per insulin type. Three dilutions for each insulin were tested: 1 mg/mL, 0.1 mg/mL, and 0.01 mg/mL. The fourth dilution in Novolog mOC87 is 0.001 mg/mL. Basaglar and Humalog blots were analyzed together on the same membrane for mOC31 in panel **A**. The circles denote which dots were used to construct **Fig. 4C-E**, which were the highest insulin concentrations tested. Aβ42 was the positive control, and TBS was the negative control. Aβ42 was diluted 1:100 in each blot unless otherwise noted.


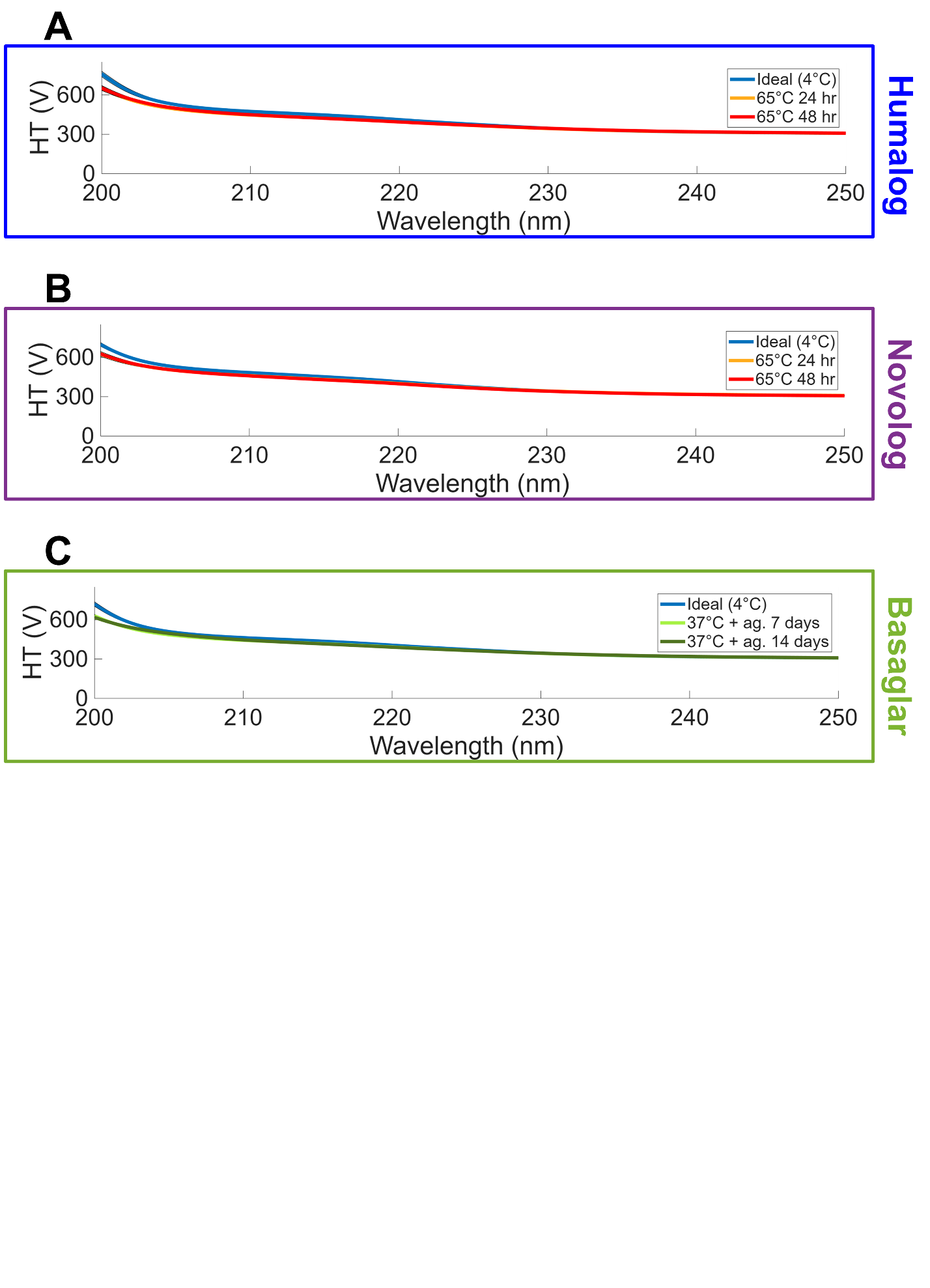


**Figure S9: High tension voltage at optimal range during circular dichroism analysis.** **(A)** Humalog, **(B)** Novolog, and **(C)** Basaglar high tension (HT) voltage across the measured wavelength range for the CD data shown in **Fig. 3**. For all spectra in this Figure, the bold line is the mean of three replicates, and the shading around each mean spectrum is ± the standard deviation. HT values remained below 600 V across most wavelengths but increased above this threshold when approaching 200 nm. Importantly, HT voltage remained well below 600 V at the key analytical wavelengths of 208, 216, and 222 nm.
